# Detection of somatic *BRCA1/2* mutations in ovarian cancer – next‐generation sequencing analysis of 100 cases

**DOI:** 10.1002/cam4.748

**Published:** 2016-05-11

**Authors:** Magdalena Koczkowska, Monika Zuk, Adam Gorczynski, Magdalena Ratajska, Marzena Lewandowska, Wojciech Biernat, Janusz Limon, Bartosz Wasag

**Affiliations:** ^1^ Department of Biology and GeneticsMedical University of GdanskGdanskPoland; ^2^ Department of PathologyMedical University of GdanskGdanskPoland; ^3^ Molecular Oncology and Genetics DepartmentInnovative Medical ForumThe Franciszek Lukaszczyk Oncology CenterBydgoszczPoland; ^4^ Department of Thoracic Surgery and TumorsLudwik Rydygier Medical College in BydgoszczNicolaus Copernicus University in TorunBydgoszczPoland

**Keywords:** *BRCA1*, *BRCA2*, next‐generation sequencing, ovarian cancer, PARP inhibitors, somatic mutation

## Abstract

The overall prevalence of germline *BRCA1/2* mutations is estimated between 11% and 15% of all ovarian cancers. Individuals with germline *BRCA1/2* alterations treated with the PARP1 inhibitors (iPARP1) tend to respond better than patients with wild‐type BRCA1/2. Additionally, also somatic *BRCA1/2* alterations induce the sensitivity to iPARP1. Therefore, the detection of both germline and somatic *BRCA1/2* mutations is required for effective iPARP1 treatment. The aim of this study was to identify the frequency and spectrum of germline and somatic *BRCA1/2* alterations in a group of Polish patients with ovarian serous carcinoma. In total, 100 formalin‐fixed paraffin‐embedded (FFPE) ovarian serous carcinoma tissues were enrolled to the study. Mutational analysis of *BRCA1*/*2* genes was performed by using next‐generation sequencing. The presence of pathogenic variants was confirmed by Sanger sequencing. In addition, to confirm the germline or somatic status of the mutation, the nonneoplastic tissue was analyzed by bidirectional Sanger sequencing. In total, 27 (28% of patient samples) mutations (20 in *BRCA1* and 7 in *BRCA2*) were identified. For 22 of 27 patients, nonneoplastic cells were available and sequencing revealed the somatic character of two *BRCA1* (2/16; 12.5%) and two *BRCA2* (2/6; 33%) mutations. Notably, we identified six novel frameshift or nonsense *BRCA1/2* mutations. The heterogeneity of the detected mutations confirms the necessity of simultaneous analysis of *BRCA1*/*2* genes in all patients diagnosed with serous ovarian carcinoma. Moreover, the use of tumor tissue for mutational analysis allowed the detection of both somatic and germline *BRCA1/2* mutations.

## Introduction

*BRCA1* and *BRCA2* tumor suppressor genes play an important role in DNA damage and repair pathways. Germline mutations in these genes are strongly associated with an increased risk of breast and ovarian cancer 1, 2. Previous studies estimated that approximately 15% of the Polish patients diagnosed with ovarian cancer carry germline *BRCA1/2* mutation 3, 4, 5. This frequency is comparable to the overall prevalence of *BRCA1/2* mutations among ovarian cancer patients worldwide 6, 7.

Although 20–70% of sporadic ovarian tumors display loss of heterozygosity (LOH) in the *BRCA1/2* loci, indicating essential role of these genes in ovarian cancer pathogenesis, somatic mutations of these genes are relatively rare finding 8, 9, 10. To date, somatic *BRCA1* mutations were reported in 5–9% of sporadic ovarian cancer cases, whereas somatic genetic variants of *BRCA2* were identified in 3–4% of tumors 11, 12, 13, 14, 15.

Recently, many clinical trials for specific therapies targeting cells with defect BRCA signaling pathway are ongoing, that is, with poly (ADP‐Ribose) polymerase 1 (PARP1) inhibitors. PARP1 is a member of chromatin‐associated polymerases involved in the single‐strand breaks repair, a common form of DNA damage 16. The clinical response rate to PARP inhibitor treatment among *BRCA1/2* mutation carriers was higher than in wild‐type patients 17, 18, 19, 20, 21. The eligibility for iPARP1 treatment is thus determined by the *BRCA1/2* mutation status, both germline and somatic. Therefore, complex mutational analysis of *BRCA1/2* genes could increase the number of patients who might benefit from PARP1 inhibitors treatment.

In this study, we established the frequency and type of *BRCA1/2* mutations. Mutational analysis of both genes was performed in 100 formalin‐fixed paraffin‐embedded tissues (FFPE) tissues from ovarian cancers.

## Materials and Methods

### Study material

In total, 100 FFPE serous ovarian carcinoma samples were enrolled to the study. All samples were obtained from the files of the Department of Pathomorphology, Medical University of Gdansk and were collected between 2008 and 2012. The histological diagnosis of ovarian serous carcinoma and the tumor tissue content (TTC%) of each sample were evaluated by pathologists. In order to obtain cancer cells from heterogeneous histological samples, tissue macrodissection was performed. The average patient age at diagnosis was 60 years (range: 36–81). Informed consent was obtained from all the patients and the research was approved by local ethics committee.

### DNA extraction

Genomic DNA was extracted from the macrodissected FFPE tissues using Cobas DNA Sample Preparation Kit (Roche, Basil, Switzerland) according to manufacturer's protocol. Quantity and quality of isolated DNA was determined with NanoDrop 1000 UV Spectrophotometer (Thermo Scientific, Canton, GA, USA) and Qubit Fluorometer (ThermoFisher Scientific, Waltham, MA, USA). In a selected 22 samples, DNA from uterus or peripheral blood samples was isolated by using Cobas DNA Sample Preparation Kit (Roche, Basilea, Switzerland) or Blood Midi kit (A&A Biotechnology, Gdynia, Poland).

### Mutational analysis

*BRCA1* and *BRCA2* mutation screening was performed using the *BRCA Tumor MASTR Plus* assay (Multiplicom, Niel, Belgium) according to the manufacturer's protocol. MiSeq targeted resequencing 99x (Illumina, San Diego, CA, USA) was performed. The read length was pair‐end and cut‐off of 4% for the Variant Allele Frequency was applied. The median coverage for all samples was 1700.

The analysis was performed with Sophia DDM software (Sophia Genetics, Saint‐Sulp). The presence of the *BRCA1/2* mutation was confirmed by bidirectional Sanger sequencing (ABI PRISM 3130, Life Technologies, Carlsbad, CA, USA). Finally, in order to determine the somatic or germline status of detected alteration in 22 *BRCA1/2* positive tumor samples, mutational analysis of DNA isolated from nonneoplastic cells was performed by PCR followed by Sanger sequencing.

## Results

**Figure 1:**
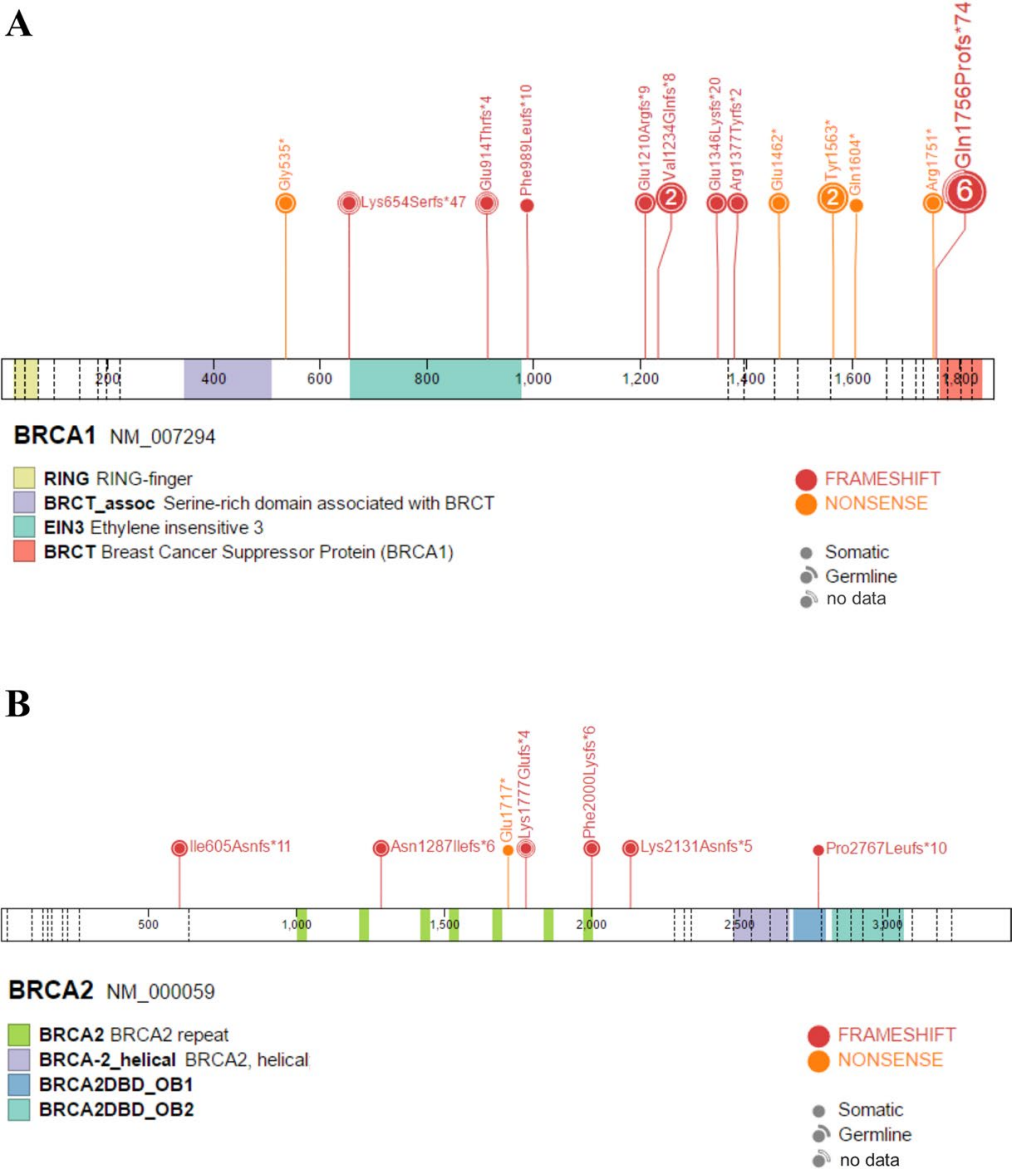
The spectrum of pathogenic mutations in the *BRCA1* (panel A) and *BRCA2* (panel B) genes in serous ovarian carcinomas using the ProteinPaint application 31. The class and origin of mutation (somatic vs. germline) is depicted with the color and shape code. Each number in circle corresponds with the total number of samples with specific alteration. Nd – no date.

From the 100 samples three (#8, #34 and #95) were excluded from the analysis due to low quality of the results. In the remaining 97 patients diagnosed with serous ovarian carcinoma, pathogenic *BRCA1/2* variants were identified in 27 (28%) samples. All genetic variants are presented at Figure 1 and Table 1. Among the *BRCA1/2* positive cases, 20 (74%) *BRCA1* and 7 (26%) *BRCA2* mutations were identified. The most frequent alteration was c.5266dupC (rs80357906, p.Gln1756Profs*74) in *BRCA1,* detected in 6.2% (*n* = 6/97) of tumors.

**Table 1:** Pathogenic mutations in the *BRCA1/2* genes in 27 serous ovarian tumors

No.	Case no	Age at diagnosis (years)	FIGO stage	Tumor content (%)	Exon/intron	Mutation in corresponding cDNA^a^	Predicted effect	Mutation type	RS Number^b^	Presence in DNA germline	% variant reads
*BRCA1*											
1	1	73	IIIB	60	11	c.2740_2759del	p.Glu914Thrfs*4	F	novel	nd	56
2	3	54	IIIB	70	20	c.5266dupC	p.Gln1756Profs*74	F	80357906	nd	51
3	6	76	IIIC	90						nd	88
4	7	64	IIIC	90						Yes	65
5	28	46	IIB	90						Yes	77
6	47	54	IIIC	75						Yes	79
7	48	58	IIIB	70						Yes	70
8	18	44	IIIB	95	16	c.4689C>G	p.Tyr1563*	N	80357433	Yes	90
9	52	39	IIIC	75						Yes	66
10	29	57	IIIB	75	11	c.3700_3704delGTAAA	p.Val1234Glnfs*8	F	80357609	Yes	87
11	32	47	IIIA	95						Yes	89
12	38	40	IIIC	55	11	c.1961delA	p.Lys654Serfs*47	F	80357522	nd	68
13	46	72	IIIC	90	11	c.2967_2970delTGTT	p.Phe989Leufs*10	F	novel	No	57
14	51	36	IIIC	95	11	c.3627dupA	p.Glu1210Argfs*9	F	80357589	Yes	58
15	53	48	IIIC	85	20	c.5251C>T	p.Arg1751*	N	80357123	Yes	87
16	68	57	IIIB	90	16	c.4810C>T	p.Gln1604*	N	80357352	No	72
17	69	59	IIIB	85	11	c.1603G>T	p.Gly535*	N	novel	Yes	82
18	84	60	IIIC	75	11	c.4035delA	p.Glu1346Lysfs*20	F	80357711	Yes	73
19	73	58	IIIC	95	14	c.4484+1G>A	r.[=,4358_4484del] p.Glu1462*	S	80358063	Yes	69
20	98	48	IIIC	75	13	c.4357+2T>G	r.[=,4186_4357del] p.Arg1377Tyrfs*2	S	80358152	Yes	74
*BRCA2*											
21	13	51	IIIB	95	11	c.5328dupT	p.Lys1777Glufs*4	F	80359500	nd	83
22	19	52	IIIB	90	11	c.5149G>T	p.Glu1717*	N	novel	No	62
23	30	62	IIIC	75	11	c.6393_6396delATTA	p.Lys2131Asnfs*5	F	397507849	Yes	84
24	36	76	IIIC	60	11	c.5993_5997dupAAGTG	p.Phe2000Lysfs*6	F	novel	Yes	59
25	58	54	IIIC	95	11	c.3860delA	p.Asn1287Ilefs*6	F	80359411	Yes	79
26	74	61	IIIC	80	18	c.8298delA	p.Pro2767Leufs*10	F	novel	No	42
27	93	53	IC	95	10	c.1813dupA	p.Ile605Asnfs*11	F	80359308	Yes	68

a Mutation type according to the Human Genome Variant Society (HGVS) nomenclature.

b A reference SNP number.

Nd, no data, F, frameshift mutation; N, nonsense mutation, S, splicing mutation.

For 22 patients with pathogenic *BRCA1/2* mutations, DNA extracted from uterus or peripheral blood leukocytes was available for further molecular studies. Within this group, only four (4.1%) mutations were found to be somatic while 18 (18.6%) were also identified in a nonneoplastic cells and were classified as germline variants. In total, 14 (78%) and four (22%) germline mutations were found in *BRCA1* and *BRCA2,* respectively.

To our knowledge, six of the 27 (22.2%) variants were not previously reported. These six novel mutations included three deletions, two substitutions, and one duplication. All these genetic variants resulted in a premature stop codon and protein truncation (Table 1).

Mutations in the *BRCA1* gene were more frequently found in patients diagnosed before or at the age of 50 compared to older individuals – 40% (8/20) versus 16% (12/77), respectively. None of the *BRCA2* alteration was identified in the group of patients younger than 50 years old. The mean age of ovarian cancer diagnosis was 54.5 (range: 36–76), 58.4 (range: 51–76) and 61.9 (range: 40–81) years for *BRCA1*,* BRCA2* and wild‐type patients, respectively.

In the study, three individuals were compound heterozygous for *BRCA1* and *BRCA2* mutations. One alteration was pathogenic and a second was previously classified as unknown variant although resulting is premature STOP codon.

Detailed histopathological and molecular data, including all variants identified in the studied group, are shown in Table S1.

## Discussion

Since personalized target therapy appears more common in modern oncology, evaluation of the highly sensitive and cost‐effective analysis accessible for routine diagnostics has become a high priority. Currently, the gold standard for detecting somatic mutations is Sanger sequencing, but its diagnostic yield is relatively low. In addition, the analysis of FFPE tumor material is challenging, because the extracted DNA is typically of poor quality and highly fragmented. Moreover, tumor cells are histologically and genetically heterogeneous and tumor DNA may be contaminated with DNA from nonneoplastic cells. Therefore, implementation of novel, powerful tools enabling the detection of low‐level mutations, such as next‐generation sequencing should be considered as a standard diagnostic procedure. The application of next‐generation sequencing allows to detect somatic mutations that may be present in a low proportion of the total DNA. In this report, we determined the frequency of somatic and germline *BRCA1/2* mutations in serous ovarian carcinoma, the entire coding sequence of these genes was analyzed by next‐generation sequencing. To distinguish true alterations from artifacts, the cut‐off of 4% for the Variant Allele Frequency was applied. The presence of pathogenic variants has been confirmed by Sanger sequencing.

Molecular analysis of *BRCA1/2* revealed the presence of pathogenic mutations in 28% of 97 studied samples. Among the *BRCA1/2* positive cases, the frequency of germline and somatic mutations was 18.6% and 4.1%, respectively. The percentage of identified somatic changes is comparable to previous reports 11, 12, 13, 14, 15, whereas the *BRCA1/2* germline mutation frequency is higher than the expected frequency in a constitutive ovarian cancer cases (~19% vs. 11–15.3%) 3, 4, 5, 6, 7. However, the explanation of this phenomena could be due to the differences in the studied patient population. In this study, all cases were classified as serous cancers which is the most frequent histological subtype of ovarian carcinomas and has the highest incidence of *BRCA1/2* mutations 7, 22 of all ovarian tumors. Not surprisingly, the majority of mutation‐positive samples (*n* = 25/27; 93%) were classified as high‐grade serous ovarian tumors. In contrast, only in two (~7%) low‐grade tumors *BRCA1/2* mutation was detected (Table 1).

According to the guidelines of the American Society of Clinical Oncology genetic testing for *BRCA1/2* mutations should be performed in each patient with ovarian cancer (1996) 23. The advantages of this approach were presented in our previous study, where more than one‐third of *BRCA1/2*‐positive patients had no family history of breast or ovarian cancer 5. Unfortunately, in this study, no information about family history of the patients was available. Another important issue is the source of the biological material used for molecular studies. In contrast to peripheral blood samples, use of a tumor tissue for mutational analysis allow to identify somatic mutations which account for additional 3–9% of patients with ovarian cancer 11, 12, 13, 14, 15. In this study, the frequency of somatic alterations was significantly higher, accounting for approximately 15% of all detected mutations.

The recommendations of the European Molecular Genetics Quality Network (EMQN) suggest to perform mutational analysis of the both *BRCA1* and *BRCA2* genes in each individual 24, which has important implications for genetic counseling, especially for cosegregation analysis. So far, the phenomenon of double *BRCA1* and *BRCA2* mutation carriers was mainly, but not only, described in the Ashkenazi Jewish population 25, 26, 27. In this study, we identified three individuals with two different germline alterations. One genetic variant was identified as pathogenic and a second was unknown variant, although resulting is premature STOP codon (Table S1). Two patients (#73 and #98) carried *BRCA1* splicing alterations (c.4484+1G>A and c.4357+2T>G) and nonsense mutations in the *BRCA2* gene (c.9976A>T; p.Lys3326*), whereas patient #47 carried *BRCA1* founder mutation (c.5266dupC; Gln1756Profs*74) and a known frameshift change in the *BRCA2* gene (c.10095delinsGAATTATATCT; p.Ser3366Asnfs*4). According to the BIC database 28, c.9976A>T change is designated as benign based on its location (the last exon of *BRCA2*) and a frequency (variant was present in 2.5% of patients and 2.1% of healthy controls). In contrast, Martin et al. (2005) described the higher frequency among patients with familial pancreatic cancer indicating its potential pathogenicity 29. c.10095delinsGAATTATATCT variant of *BRCA2* was previously detected in two Polish patients with familial aggregation of the disease 30. In both individuals, the presence of other *BRCA1*/*2* mutations was excluded. Finally, in silico analysis suggest a possible impact of c.10095delinsGAATTATATCT on mRNA splicing.

Finally, to our knowledge six presumably novel mutations in the *BRCA1* and *BRCA2* genes were identified (Table 1). These included two nonsense and four frameshift variants, all resulting in premature termination of translation.

In conclusion, the application of a targeted assay using next‐generation sequencing of FFPE samples allowed the identification both somatic and germline *BRCA1/2* mutations in a group of patients with serous ovarian tumors. As we previously mentioned, the routine genetic screening for at least five recurrent *BRCA1* mutations (c.5266dup, c.181T>G, c.3700_3704del, c.68_69delAG, and c.4034delA) in all ovarian cancer patients in the Polish population should be performed 30. However, the use of a tumor sample for molecular analysis and further mutational analysis of the entire coding sequence of both *BRCA1* and *BRCA2* may significantly increase the total number of patients who may potentially benefit from targeted therapies with PARP inhibitors. In addition, such a genetic testing would allow to identify more family members with *BRCA1/2* mutations.

## Conflict of Interest

L.J. did receive honoraria for advisory activities from AstraZeneca. W.B. did receive honoraria for advisory and consulting functions and educational activities from AstraZeneca.

## Supporting information


**Table S1.** All identified variants in the *BRCA1/2* genes in the serous ovarian tumors.
