# Supplementary material for: Detection of somatic BRCA1/2 mutations in ovarian cancer – next‐generation sequencing analysis of 100 cases
Source: Cancer Med. 2016 May 11;5(7):1640–6. doi: 10.1002/cam4.748 (PMC4867663; doi:10.1002/cam4.748)
Supplement: Supplementary file 1 — Table S1. All identified variants in the BRCA1/2 genes in the serous ovarian tumors. [file CAM4-5-1640-s001.pdf]

TABLE S1. All identified variants in the *BRCA1/2* genes in the serous ovarian tumors.

Case No.	Tumor content (%)	***BRCA1*** Mutation in corresponding cDNA[a]	Predicted effect	Mutation type[b]	RS Number[c]	% variant reads	***BRCA2*** Mutation in corresponding cDNA[a]	Predicted effect	Mutation type[b]	RS Number[c]	% variant reads
1	60	c.2740_2759del	p.Glu914Thrfs*4	F	novel	56	-	-	-	-	-
2	90	-	-	-	-	-	c.5744C>T	p.Thr1915Met	M	4987117	84
3	70	c.5266dupC	p.Gln1756Profs*74	F	80357906	51	-	-	-	-	-
4	50	-	-	-	-	-	-	-	-	-	-
5	85	c.5095C>T	p.Arg1699Trp	M	55770810	70	-	-	-	-	-
6	90	c.5266dupC	p.Gln1756Profs*74	F	80357906	88	c.5744C>T	p.Thr1915Met	M	4987117	53
7	90	c.5266dupC	p.Gln1756Profs*74	F	80357906	65	-	-	-	-	-
9	70	c.5056C>G<br>c.1067A>G	p.His1686Asp<br>p.Gln356Arg	M<br>M	45517388<br>1799950	61<br>80	-	-	-	-	-
10	80	-	-	-	-	-	-	-	-	-	-
11	90	-	-	-	-	-	-	-	-	-	-
12	10	c.2077G>A	p.Asp693Asn	M	4986850	47	c.10095delinsGAATTATATCT	p.Ser3366Asnfs*4	F	27617480	42
13	95	-	-	-	-	-	c.5328dupT	p.Lys1777Glufs*4	F		83
14	80	-	-	-	-	-	-	-	-	-	-
15	40	-	-	-	-	-	c.2971A>G<br>c.2229T>C<br>c.1365A>G<br>c.865A>C	p.Asn991Asp<br>p.(=)<br>p.(=)<br>p.Asn289His	M<br><br><br>M	1799944<br>1801499<br>1801439<br>766173	62
16	35	-	-	-	-	-	c.5199C>T	p.(=)		28897734	51
17	90	-	-	-	-	-	-	-	-	-	-
18	95	c.4689C>G	p.Tyr1563*	N	80357433	90	-	-	-	-	-
19	90	-	-	-	-	-	c.5149G>T	p.Glu1717*	N	novel	62
20	70	-	-	-	-	-	-	-	-	-	-
21	80	-	-	-	-	-	-	-	-	-	-
22	75	c.1067A>G	p.Gln356Arg	M	1799950	53	c.6796A>G	p.Asn2266Asp	M	novel	17
23	65	-	-	-	-	-	-	-	-	-	-
24	25	c.1067A>G	p.Gln356Arg	M	1799950	46	-	-	-	-	-
25	75	c.1067A>G	p.Gln356Arg	M	1799950	92	-	-	-	-	-
26	95	c.3119G>A	p.Ser1040Asn	M	4986852	35	-	-	-	-	-
27	60	-	-	-	-	-	-	-	-	-	-
28	90	c.5266dupC	p.Gln1756Profs*74	F	80357906	77	-	-	-	-	-
29	75	c.3700_3704delGTAAA	p.Val1234Glnfs*8	F	80357609	87	-	-	-	-	-
30	75	-	-	-	-	-	c.6393_6396delATTA	p.Lys2131Asnfs*5	F	397507849	84
31	99	c.1067A>G	p.Gln356Arg	M	1799950	58	c.2971A>G	p.Asn991Asp	M	1799944	56
32	95	c.3700_3704delGTAAA	p.Val1234Glnfs*8	F	80357609	87	-	-	-	-	-
33	95	-	-	-	-	-	c.917C>A	p.Phe3090Leu	M	novel	50

35	55	-	-	-	-	-	-	-	-	-	-
36	60	-	-	-	-	-	c.5993_5997dupAAGTG	p.Phe2000Lysfs*6	F	novel	59
37	35	-	-	-	-	-	-	-	-	-	-
38	55	c.1961delA	p.Lys654Serfs*47	F	80357522	68	c.9976A>T	p.Lys3326*	N	11571833	68
39	85	-	-	-	-	-	-	-	-	-	-
40	30	-	-	-	-	-	c.5744C>T	p.Thr1915Met	M	4987117	66
41	95	-	-	-	-	-	-	-	-	-	-
42	65	-	-	-	-	-	c.5455C>T	p.Pro1819Ser	M	80358768	50
43	95	c.4956G>A	p.Met1652Ile	M	1799967	50	-	-	-	-	-
44	25	c.4956G>A	p.Met1652Ile	M	1799967	50	-	-	-	-	-
45	95	-	-	-	-	-	c.8182G>A	p.Val2728Ile	M	2889749	33
46	90	c.2967_2970delTGTT	p.Phe989Leufs*10	F	novel	57	-	-	-	-	-
47	75	c.5266dupC	p.Gln1756Profs*74	F	80357906	79	c.10095delinsGAATTATATCT	p.Ser3366Asnfs*4	F	27617480	74
48	70	c.5266dupC	p.Gln1756Profs*74	F	80357906	70	-	-	-	-	-
49	80	-	-	-	-	-	c.978C>A	p.Ser326Arg	M	28897706	79
50	90	-	-	-	-	-	c.1788T>C	p.(=)		11571642	18
51	95	c.3627dupA	p.Glu1210Argfs*9	F	80357589	58	-	-	-	-	-
52	75	c.4689C>G	p.Tyr1563*	N	80357433	90	-	-	-	-	-
53	85	c.5251C>T	p.Arg1751*	N	80357123	87	-	-	-	-	-
54	95	-	-	-	-	-	c.8182G>A	p.Val2728Ile	M	2889749	11
55	55	c.1067A>G	p.Gln356Arg	M	1799950	27	-	-	-	-	-
56	50	-	-	-	-	-	c.5070A>C	p.Lys1690Asn	M	56087561	43
57	95	-	-	-	-	-	-	-	-	-	-
58	95	-	-	-	-	-	c.3860delA	p.Asn1287Ilefs*6	F	80359411	79
59	30	c.2077G>A<br>c.1067A>G	p.Asp693Asn<br>p.Gln356Arg	M<br>M	4986850<br>1799950	46<br>43	-	-	-	-	-
60	90	-	-	-	-	-	-	-	-	-	-
61	75	-	-	-	-	-	-	-	-	-	-
62	70	-	-	-	-	-	-	-	-	-	-
63	55	c.4641G>T	p.Leu1547Phe	M	novel	13	-	-	-	-	-
64	90	-	-	-	-	-	-	-	-	-	-
65	90	c.1067A>G	p.Gln356Arg	M	1799950	92	c.5744C>T	p.Thr1915Met	M	4987117	91
66	90	-	-	-	-	-	-	-	-	-	-
67	65	c.591C>T	p.(=)		1799965	68	-	-	-	-	-
68	90	c.4810C>T	p.Gln1604*	N	80357352	72	-	-	-	-	-
69	85	c.1603G>T	p.Gly535*	N	novel	82	-	-	-	-	-
70	60	-	-	-	-	-	-	-	-	-	-
71	80	-	-	-	-	-	-	-	-	-	-
72	95	-	-	-	-	-	-	-	-	-	-
73	95	c.4484+1G>A	r.[=,4358_4484del]<br>p.Glu1462*	S	80358063	69	c.9976A>T	p.Lys3326*	N	11571833	68
74	80	-	-	-	-	-	c.8298delA	p.Pro2767Leufs*10	F	novel	42

75	95	c.4956G>A	p.Met1652Ile	M	1799967	13	c.4068G>A	p.(=)		28897724	85
76	95	-	-	-	-	-	-	-	-	-	-
77	75	c.1708C>A	p.Pro570Thr	M	novel	34	-	-	-	-	-
78	90	-	-	-	-	-	-	-	-	-	-
79	90	c.1067A>G	p.Gln356Arg	M	1799950	21	c.5070A>C	p.Lys1690Asn	M	56087561	73
80	95	c.1067A>G	p.Gln356Arg	M	1799950	86	-	-	-	-	-
81	70	c.1067A>G	p.Gln356Arg	M	1799950	43	-	-	-	-	-
82	85	-	-	-	-	-	c.7544C>T	p.Thr2515Ile	M	28897744	67
83	95	-	-	-	-	-	-	-	-	-	-
84	75	c.4035delA	p.Glu1346Lysfs*20	F	80357711	73					
85	95	-	-	-	-	-	-	-	-	-	-
86	85	c.4956G>A	p.Met1652Ile	M	1799967	25	-	-	-	-	-
87	70	-	-	-	-	-	-	-	-	-	-
88	90	c.1385G>A<br>c.767G>A	p.Gly462Glu<br>p.Arg256Lys	M<br>M	novel<br>novel	15<br>11	-	-	-	-	-
89	95	c.3119G>A	p.Ser1040Asn	M	4986852	19	-	-	-	-	-
90	95	-	-	-	-	-	c.10154G>A<br>c.8851G>A	p.Arg3385His<br>p.Ala2951Thr	M<br>M	80358398<br>11571769	15<br>14
91	99	-	-	-	-	-	c.4068G>A	p.(=)		28897724	85
92	85	-	-	-	-	-	-	-	-	-	-
93	95	-	-	-	-	-	c.1813dupA	p.Ile605Asnfs*11	F	80359308	68
94	80	c.1067A>G	p.Gln356Arg	M	1799950	24	-	-	-	-	-
96	70	-	-	-	-	-	-	-	-	-	-
97	90	-	-	-	-	-	-	-	-	-	-
98	75	c.4357+2T>G	r.[=,4186_4357del]<br>p.Arg1377Tyrfs*2	S	80358152	74	c.9976A>T	p.Lys3326*	N	11571833	74
99	80	-	-	-	-	-	-	-	-	-	-
100	60	-	-	-	-	-	-	-	-	-	-

[a] Mutation type according to the HGVS nomenclature; HGVS, Human Genome Variant Society. [b] F – frameshift variant; N – nonsense variant; M – missense variant; S – splicing variant. [c] A reference SNP number
